# Evidence of Racial and Geographic Disparities in the Use of Medicare Observation Stays and Subsequent Patient Outcomes Relative to Short-Stay Hospitalizations

**DOI:** 10.1089/heq.2017.0055

**Published:** 2018-04-01

**Authors:** Brad Wright, Xuan Zhang, Momotazur Rahman, Mahshid Abir, Padmaja Ayyagari, Keith E. Kocher

**Affiliations:** ^1^ Department of Health Management and Policy, University of Iowa, Iowa City, Iowa.; ^2^ Public Policy Center, University of Iowa, Iowa City, Iowa.; ^3^ Department of Economics, Brown University, Providence, Rhode Island.; ^4^ Department of Health Services, Policy, and Practice, Brown University, Providence, Rhode Island.; ^5^ Department of Emergency Medicine, University of Michigan, Ann Arbor, Michigan.; ^6^ RAND Corporation, Santa Monica, California.; ^7^ Institute for Healthcare Policy and Innovation, University of Michigan, Ann Arbor, Michigan.; ^8^ Center for Healthcare Outcomes and Policy, University of Michigan, Ann Arbor, Michigan.

**Keywords:** disparities, hospitals, Medicare, observation, outcomes

## Abstract

**Purpose:** To examine racial and geographic disparities in the use of—and outcomes associated with—Medicare observation stays versus short-stay hospitalizations.

**Methods:** We used 2007–2010 fee-for-service Medicare claims, including 3,555,994 observation and short-stay hospitalizations for individuals over age 65. We estimated linear probability models with hospital fixed effects to identify within-facility disparities in observation stay use, estimated in-hospital mortality, 30- and 90-day postdischarge mortality, return emergency department (ED) visits, and hospital readmissions as a function of placement in observation using linear probability models, propensity-score matching, and interaction terms.

**Results:** We identified racial and geographic disparities in the likelihood of observation stay use within hospitals (blacks 3.9% points more likely than whites, rural 5.4% points less likely than urban). Observation is associated with an increased likelihood of returning to the ED within 30 or 90 days and a decreased likelihood of readmission or mortality, but there are racial and geographic disparities in these outcomes.

**Conclusion:** While observation generally results in improved outcomes, disparities in these outcomes and the use of observation stays within hospitals are concerning and may be driven by clinical and nonclinical factors.

## Introduction

In 2011, nearly 20.4 million individuals over age 65 visited an emergency department (ED).^1^ Approximately 7 million of these individuals were admitted to the hospital, and 1.5 million others were held for observation—a hospital-based outpatient service used for evaluation and treatment until a decision is made regarding inpatient admission or discharge.^2^ Proponents of observation stays argue that they afford providers additional time to make accurate diagnoses and treatment decisions and represent a cost-effective substitute for short-stay hospitalizations.^3–6^ However, critics counter that observation stays shift the high cost of inpatient care to patients, because these stays resemble inpatient care, but are billed as outpatient care.^7^

Medicare beneficiaries are increasingly being held for observation rather than admitted,^8^ and there is also evidence of racial and geographic disparities in the use of observation stays,^9,10^ which may be driven by differences in patient characteristics or hospital-specific factors.^11–14^ Therefore, it is important to determine the extent to which racial and geographic disparities in the use of observation stays are driven by differences within or between hospitals and the extent to which these disparities translate into differences in patient outcomes. Using Medicare claims, we compared disparities in the assignment of observation versus short-stay hospitalization by race and rurality. We then evaluated differences in in-hospital mortality, 30- and 90-day postdischarge mortality, return ED visits, and hospital readmissions.

## Methods

Using years 2007–2010 of the 100% Medicare Inpatient and Institutional Outpatient Research Identifiable Claims Files and the Medicare Enrollment File, we generated a sample of individuals over age 65, enrolled in fee-for-service Medicare, with at least one observation stay or short-stay hospitalization (≤2 days) in a given year. We identified observation stays using any one of four combinations of revenue center codes (0760 or 0762) and Healthcare Common Procedure Coding System codes (G0378 or G0379). Using admission and discharge dates from the inpatient claims, we identified patients with a short-stay hospitalization. We excluded patients whose observation stay was converted to an inpatient admission, because we could not clearly categorize them. To ensure comparability between groups, we also excluded long observation stays (>48 h).

First, we generated descriptive statistics for our sample stratified by whether the individual had a short-stay hospitalization, observation stay, or both during a given year. We determined mortality, readmission, and return ED visit rates based on all annual events, while the remaining demographic characteristics were based on individuals' first event during the year to avoid high utilizers skewing the data.

Then, we modeled placement under observation (vs. short-stay hospitalization) as a function of race, rurality, age, gender, and comorbid conditions (using the modifications to the Charlson Comorbidity Index^15^ suggested by Quan et al.^16^). We also adjusted for seasonality, weekend admissions, and secular time trends in observation stay use. Next, we included hospital fixed effects, which account for all time-invariant hospital specific factors that influence observation stays and allow us to determine whether racial minority and/or rural patients are more likely to be placed under observation *within* hospitals. Because nonlinear models failed to converge with the inclusion of over 4744 hospital fixed effects, we estimated linear probability models and clustered standard errors at the hospital level to account for correlated data within facilities. To account for individuals being included in our short-stay hospitalization sample solely because they died within 48 h, we conducted a sensitivity analysis with 399,777 cases of in-hospital mortality removed, and our results were consistent.

Next, we adapted the methods of Jha et al. to characterize the hospitals in our study into four groups, representing all combinations of high and low “observation hospitals” and high and low “short-stay hospitals.”^12,13^ We defined “high observation hospitals” as those with an observation stay rate above the sample average and “low observation hospitals” as those with an observation stay rate at or below the sample average. Similarly, we defined “high short-stay hospitals” and “low short-stay hospitals” using the short-stay hospitalization rate relative to the sample average. For each hospital type, we used weighted averages to calculate the proportion of patients who were black and the proportion of patients who were rural residents.

Then, we estimated several linear probability models to evaluate differences in outcomes as a function of observation stays relative to short-stay hospitalizations. Again, we opted for linear probability models because nonlinear models failed to converge with the inclusion of our 4744 hospital fixed effects. In particular, we examined four outcomes as follows: in-hospital and postdischarge mortality, return ED visits, and readmissions. We identified postdischarge mortality, return ED visits, and readmissions if they occurred at least once at any hospital within 30 or 90 days. These outcomes are inversely related to hospital quality,^17^ and unplanned returns to the hospital are a costly source of avoidable healthcare expenditures.^18^ In each analysis, events occurring within 30 or 90 days of the end of our study period were excluded as index events.

We generated propensity scores using a logistic regression model based on patient age, gender, race/ethnicity, rurality of residence, discharge location, Quan score for comorbid conditions, seasonality, weekend admission, and year. The nearest neighbor method was used, and we visually inspected the propensity score distribution to confirm that the two groups were well balanced. Since individuals could have multiple stays in a year, the analysis was at the person-event level with clustered standard errors. We excluded individuals who died in the hospital or were transferred within 48 h or 2 days from our models of postdischarge outcomes, as this may have artificially truncated an otherwise longer stay.

As a sensitivity analysis, we repeated our propensity-score matching approach, but limited our sample to individuals with a discharge diagnosis of chest pain to further reduce heterogeneity between groups. Chest pain is an important bellwether condition, amenable to treatment in both settings, and the most common reason for use of observation services.^5,8,19,20^ The results were similar, except that mortality in chest pain patients is more comparable between observation and short-stay hospitalization patients than it is for all diagnoses. These results are available in Appendix Table A1.

Finally, we examined racial and geographic disparities in the relationship between placement under observation and patient outcomes. We estimated the same linear probability models on an unmatched sample, including the matching variables as covariates and adding interaction terms among observation, race, and rurality of residence. We also included hospital fixed effects to adjust for variation in our outcomes between facilities. The full results are available in Appendix Tables A2–A5. This study was approved by the University of Iowa IRB.

## Results

Descriptive statistics are shown in Table 1. Given the large sample size, all comparisons are statistically significant, but there are few meaningful differences between groups. For example, there are small notable differences in regional use of observation stays. In addition, individuals with a short-stay hospitalization appeared to be in slightly worse health than the observation stay group, based on their Quan score. Rates of readmission and return ED visits were significantly higher among individuals who experienced both an observation stay and a short-stay hospitalization during the year, likely reflecting that these individuals had two or more visits by definition.

**Table 1.** **Description of Beneficiaries from Emergency Department Visit in a Year**

	Short stay	Observation stay	Both
30-Day mortality rate (%)	4.22	1.72	2.29
90-Day mortality rate (%)	7.58	4.26	5.94
30-Day readmission rate (%)	13.97	8.98	24.77
90-Day readmission rate (%)	25.28	18.36	46.05
30-Day return ED visit rate (%)	9.66	11.16	26.79
90-Day return ED visit rate (%)	19.47	22.31	48.80
Age (in years)	77.48	77.86	78.01
Male (%)	43.93	37.64	41.14
Black (%)	8.93	8.58	10.45
White (%)	86.53	86.99	85.31
Other race (%)	4.43	4.32	4.15
% Rural	23.94	25.33	26.55
Quan Index	0.93	0.67	1.03
Midwest	27.20	28.29	30.68
Northeast	18.79	14.83	13.51
South	37.93	42.31	40.57
West	16.07	14.57	15.25
Weekend	22.06	24.65	23.25
Spring	28.44	27.93	28.14
Fall	20.10	20.42	20.86
Winter	23.84	23.38	22.35
*n*	1,499,692	1,612,776	239,375

Source: Authors' analysis of Medicare claims data, 2007–2010.

A chi-squared test rejects the null hypothesis that the means are equal in all of the three groups for each variable. A pairwise *t*-test confirms that all the means are statistically different (*p*<0.01), except for the difference in age between the second and the third group.

ED, emergency department.

**FIG. 1.**
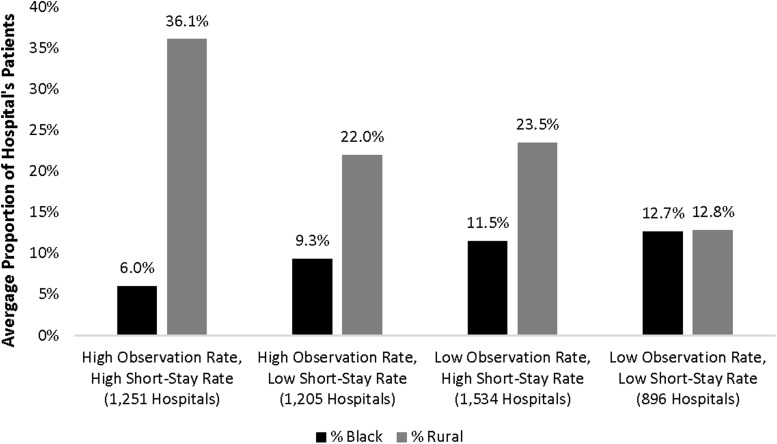
Patient race and rurality by hospital propensity to use observation versus short-stay hospitalization. Source: Authors' analysis of Medicare claims data, 2007–2010.

Figure 1 presents disparities in observation use across hospitals, showing that black and rural patients are disproportionately clustered according to the hospital's relative use of observation stays and short-stay hospitalizations. Hospitals with above average rates of both observation stays and short-stay hospitalizations tend to serve a patient population with fewer blacks and more rural residents. By contrast, hospitals with below average rates of both observation stays and short-stay hospitalizations tend to serve a patient population with more than double the proportion of blacks and nearly one-third the proportion of rural residents.

Table 2 presents the results of our models predicting placement under observation, with and without hospital fixed effects. Overall, we find that—compared to whites—blacks are slightly more likely to be placed under observation, while those of other races are slightly less likely to be placed under observation. However, our hospital fixed effects model finds substantial within-hospital racial disparities in observation stay use. In particular, blacks are 3.9% points more likely to be placed under observation, and those of other races are 2.1% points more likely to be placed under observation, than whites at the same facility. Similarly, we find no overall association between rural residence and the likelihood of placement under observation. However, within a given hospital, rural patients are 5.4% points less likely to be placed under observation than their urban counterparts. The remaining coefficients are similar across both models, indicating that—to the extent that these factors predict observation use—their influence is comparable between and within hospitals. For example, women are more likely than men to be placed under observation, sicker individuals are more likely to be admitted than observed, and there is a clear shift from short-stay hospitalizations to observation stays over time.

**Table 2.** **Marginal Effects on Assignment to Observation Care**

Variables	Percentage point change	Percentage point change
Black	0.53^a^ (0.093)	3.91^a^ (0.152)
Other race	−0.78^a^ (0.133)	2.12^a^ (0.187)
Rural residence	0.043 (0.060)	−5.43^a^ (0.245)
Age (in years)	0.017^a^ (0.003)	−0.006 (0.006)
Female	6.22^a^ (0.054)	5.32^a^ (0.069)
Quan score	−4.03^a^ (0.018)	−3.76^a^ (0.051)
Weekend	2.50^a^ (0.062)	1.87^a^ (0.091)
Spring	−1.27^a^ (0.070)	−1.33^a^ (0.100)
Fall	0.206^a^ (0.078)	0.221^b^ (0.104)
Winter	−1.43^a^ (0.074)	−1.52^a^ (0.114)
2008	42.8^a^ (0.197)	37.3^a^ (0.770)
2009	46.2^a^ (0.197)	40.4^a^ (0.751)
2010	48.3^a^ (0.198)	42.6^a^ (0.736)
Constant	0.596 (0.322)	9.57^a^ (0.854)
Observations	3,555,994	3,555,994
R-squared	0.021	0.022
Hospital FE	No	Yes (*n*=4744)

Source: Authors' analysis of Medicare claims data, 2007–2010.

^a^ *p*<0.01.

^b^ *p*<0.05.

FE, fixed effects.

Table 3 presents the results of our models predicting patient outcomes as a function of observation placement among our propensity score matched sample. Compared to patients with a short-stay hospitalization, patients placed under observation were 9.6% points less likely to die in the hospital, 1.9% points less likely to die within 30 days postdischarge, and 2.2% points less likely to die within 90 days postdischarge. Similarly, patients placed under observation were 3.4% points less likely than those with a short-stay hospitalization to be readmitted within 30 days postdischarge and 4.2% points less likely to be readmitted within 90 days. By contrast, patients placed under observation were 1% point more likely than those with a short-stay hospitalization to return to the ED within 30 days postdischarge and 2.1% points more likely to return to the ED within 90 days. It is helpful to consider these marginal effects relative to the sample mean for each outcome. For example, the 3.4% point reduction in 30-day readmissions represents a 27.6% reduction relative to the sample mean of 12.3.

**Table 3.** **Outcomes Associated with Observation Versus Short-Stay Hospitalization Among Medicare Beneficiaries (Percentage Point Change)**

	Return ED visit		Readmission		Mortality		
	30-Day	90-Day	30-Day	90-Day	In-event	30-Day	90-Day
Observation stay	1.0^a^ (0.04)	2.1^a^ (0.05)	−3.4^a^ (0.04)	−4.2^a^ (0.05)	−9.6^a^ (0.02)	−1.9^a^ (0.02)	−2.2^a^ (0.03)
% Change relative to sample mean	8.6	9.1	−27.6	−17.9	−174.5	−65.5	−37.3
Sample mean (SD)	11.6 (32.1)	23.0 (42.1)	12.3 (32.9)	23.4 (42.3)	5.5 (22.8)	2.9 (16.7)	5.9 (23.5)
Model observations	3,075,568	3,013,941	3,089,282	3,058,910	3,554,626	3,155,052	3,155,052
Sample observations	3,266,206	3,200,857	3,280,835	3,248,710	3,769,293	3,350,604	3,350,604

Source: Authors' analysis of Medicare claims data, 2007–2010.

Standard errors in parentheses.

^a^ *p*<0.01.

Table 4 presents the disparate marginal effects of observation placement on our outcomes by race and rurality. Full model results are available in Appendix Tables A3–A5. Overall, we find that regardless of the direction of the association, placement under observation has a more pronounced influence among nonwhite races versus whites. For instance, placement under observation is associated with a larger increase in the likelihood of returning to the ED within 30 or 90 days among blacks and those of other races than among whites. Similarly, placement under observation is associated with a larger decrease in the likelihood of 30- or 90-day readmission and in-event mortality among blacks and those of other races than whites. Only for 30- or 90-day postdischarge mortality did placement under observation have a more pronounced—although small—association among whites than blacks and those of other races.

**Table 4.** **Disparities in Outcomes Associated with Observation Versus Short-Stay Hospitalization Among Medicare Beneficiaries (Percentage Point Change)**

	Return ED visit		Readmission		Mortality		
	30-Day	90-Day	30-Day	90-Day	In-event	30-Day	90-Day
Nonrural							
White	0.5	1.0	−3.4	−4.3	−9.9	−1.2	−1.5
Black	1.0	1.9	−4.4	−6.0	−10.5	−0.8	−1.1
Other race	1.2	1.9	−4.7	−6.6	−10.3	−0.8	−1.1
Rural							
White	−0.6	−0.5	−3.8	−4.6	−10.2	−1.2	−1.6
Black	−0.1	0.4	−4.8	−6.3	−10.8	−0.8	−1.2
Other race	0.1	0.4	−5.1	−6.9	−10.6	−0.8	−1.2

Source: Authors' analysis of Medicare claims data, 2007–2010.

The association of observation with our outcomes also varies by rurality, which can be calculated by subtracting the treatment effects for a given race between the rural and nonrural rows of Table 4. For example, among rural residents, placement under observation is associated with an additional 1.1% point decrease in 30-day return ED visits and an additional 1.5% point decrease in 90-day return ED visits. In some cases, this means that the difference between observations is being associated with an increase or a decrease in return ED visits. Among rural residents, placement under observation is also associated with an additional 0.4% and 0.3% point decrease in 30-and 90-day readmissions, respectively. In short, rural patients placed under observation are less likely to return to the hospital than urban patients. Rural/urban disparities were less pronounced for mortality. When associations were detected, they were small and suggested that rural patients placed under observation were slightly less likely to die during the index event or within 90 days postdischarge than urban patients. There was no difference in 30-day mortality.

## Discussion

As Medicare observation stay use grows, understanding the impact of observation stays on patient outcomes is increasingly important. We found little evidence that observation stays are associated with worse outcomes than short-stay hospitalizations. Overall, patients placed under observation are slightly more likely to return to the ED within 30- or 90 days postdischarge, but much less likely to be readmitted to the hospital within 30- or 90 days postdischarge or die during their hospital stay or within 30- or 90 days postdischarge. These findings parallel those of recent studies, which found that patients placed under observation were less likely than admitted patients to die or return to the hospital within 30 days.^21–23^ However, we are the first to document significant racial and geographic disparities in both the use of observation stays relative to short-stay hospitalizations and the outcomes associated with placement under observation.

Disparities in observation stay use might be related to which hospitals individuals visit for care. For example, prior research found significant variation in observation stay use between hospitals,^9,10^ including evidence that hospitals serving a larger proportion of black patients are less likely to provide any observation stays, and those that do have a lower conditional prevalence of observation stays.^24^ Similarly, we found that black patients disproportionately seek care at hospitals with low observation and short-stay hospitalization rates, while rural patients disproportionately seek care at hospitals with high observation and short-stay hospitalization rates. This suggests that black patients are less likely to be placed in observation, while rural patients are more likely to be placed in observation. However, we also found that—within any given hospital—blacks and other nonwhite patients are significantly more likely to be placed under observation than whites, and rural residents are significantly less likely to be placed under observation than nonrural patients. Thus, in contrast to numerous studies that suggest disparities result from vulnerable patients disproportionately receiving care in low-quality hospitals,^11–13,25^ our findings indicate that racial and geographic disparities persist regardless of where individuals go for care. This is consistent with evidence of within-hospital racial disparities in the disposition of chest pain patients in the ED^26^ and ED length of stay for admitted patients.^27^

We also find evidence of racial and geographic disparities in postobservation outcomes. Relative to short-stay hospitalizations, observation stays are more likely to result in return ED visits for racial minorities—especially blacks—than whites. However, the opposite is true for readmissions, where rates are lower for racial minorities than whites following an observation stay. Similarly, we found disparities between rural and nonrural patients, with nonrural patients being more likely to return to the ED and/or be readmitted following an observation stay. We did not observe significant racial or geographic disparities in mortality related to observation use.

We can only speculate about the possible causes of these disparities. First, the increased likelihood of minority and nonrural patients being placed in observation may be related to nonclinical needs influencing medical care decisions. When making disposition decisions, ED providers often consider the patient's perceived social needs and the availability of follow-up options within the local healthcare system.^28,29^ The extent to which providers trust patients may also play a role in this decision making process.^30,31^ For example, if nonwhite race or urbanity is associated with factors such as income, social and family support, and decreased access to resources like transportation, providers may keep these patients in observation, whereas they might discharge individuals whom they feel can arrange timely follow-up appointments or return quickly to the ED if their clinical status changes. Variation in physician practice patterns drives significant variations in care delivery between and within hospitals,^32^ but the patient's role should not be ignored. Physician–patient communication studies suggest that white patients may ask to be admitted or demand to go home more assertively than nonwhite patients.^33^

Second, these disparities may reflect implicit bias among providers.^34^ For example, observation patients are known to receive fewer services than patients with a short-stay hospitalization.^22^ Thus, to the extent that inpatient admission is considered preferable to observation, providers implicitly biased against nonwhite patients may observe—rather than admit—them. Such implicit biases could also explain the use of observation stays as a response to an individual's perceived nonclinical needs as described above. For example, providers may make assumptions about a patient's ability to coordinate follow-up care based on race.^35^

Third and finally, this pattern may simply reflect a difference in underlying clinical needs not adequately accounted for by our models. For example, if minority and/or nonrural patients generally present to the ED in worse health than white and/or rural patients, they may require a period of observation or longer inpatient hospitalization, whereas the white and/or rural patients may be treated and released directly from the ED or admitted for a short-stay hospitalization.

### Limitations

Our study may be limited by unobserved differences between the observed and admitted patients in our sample, which we cannot account for using claims data, despite using propensity score matching. However, we are encouraged by the robust findings from our sensitivity analysis among chest pain patients and evidence that observation and short-stay hospitalization patients share similar characteristics.^36^ Of course, not all Medicare beneficiaries had an observation stay or short-stay hospitalization during our study period, potentially limiting the generalizability of our findings. Moreover, because propensity score matching yields an average treatment effect, we cannot conclude how any particular individual would be affected by observation. Finally, using billing codes to identify observation stays precludes differentiation of protocol-driven observation units from other observation stays with less robust evidence of effectiveness.^5^

## Conclusion

Our study demonstrates that practice patterns related to observation stays are not immune to similar disparities documented in other areas of emergency care delivery.^35^ Generally, however, we find that observation stays result in improvements in the outcomes we measured. Nevertheless, there are important tradeoffs by race and rurality in outcomes such as ED return visits and hospital readmissions. We also found racial and geographic disparities in observation use within hospitals, which raises questions about the extent of disparities in treatment between the observation and inpatient settings and highlights the importance of determining the causes of those disparities in future work. Reducing variation in care delivery using evidence-based guidelines is one way to reduce disparities in the ED.^35^ Consequently, racial and geographic disparities may be more pronounced in hospitals without a protocol-driven observation unit. We lacked the data necessary to investigate this, but it remains an important question for future research.

## Abbreviation Used

ED: emergency department

## Appendix

**Appendix Table A1.** **Outcomes Associated with Observation Versus Short-Stay Inpatient Hospitalization Among Medicare Beneficiaries with Chest Pain (Percentage Point Change)**

	Return ED visit		Readmission		Mortality		
	30-Day	90-Day	30-Day	90-Day	In-event	30-Day	90-Day
Observation stay	0.5^a^ (0.08)	1.3^a^ (0.1)	−3.8^a^ (0.07)	−5.1^a^ (0.1)	−1.3^a^ (0.02)	−0.3^a^ (0.02)	−0.6^a^ (0.04)
% Change relative to sample mean	4.8	6.1	−42.2	−28.0	−216.7	−50.0	−31.6
Model observations	704,966	699,348	706,130	703,815	747,630	708,361	708,361
Sample observations	748,901	742,966	750,142	747,695	793,765	752,506	752,506
Sample mean (SD)	10.4 (30.5)	21.3 (40.9)	9.0 (28.6)	18.2 (38.6)	0.6 (7.5)	0.6 (7.9)	1.9 (13.7)

Standard errors in parentheses.

^a^ *p*<0.01.

ED, emergency department.

**Appendix Table A2.** **Linear Probability Model of In-Hospital Mortality**

Variables	Percentage point change
Observation	−9.88^a^ (0.109)
Observation×black	−0.667^a^ (0.184)
Observation×other race	−0.431^b^ (0.230)
Observation×rural residence	−0.259^b^ (0.148)
Black	0.669^a^ (0.145)
Other race	0.458^a^ (0.171)
Rural residence	1.24^a^ (0.124)
Age (in years)	0.305^a^ (0.0032)
Female	−0.440^a^ (0.0264)
Quan Index	1.49^a^ (0.0199)
Weekend	1.67^a^ (0.0361)
Spring	0.491^a^ (0.0322)
Fall	0.387^a^ (0.0357)
Winter	1.17^a^ (0.0365)
2008	0.303 (0.793)
2009	−0.0495 (0.794)
2010	−0.399 (0.794)
Constant	−16.0^a^ (0.828)
Observations	3,555,994
Hospital fixed effects	*n*=4744

^a^ *p*<0.01.

^b^ *p*<0.05.

**Appendix Table A3.** **Linear Probability Model of Return Emergency Department Visits (Percentage Point Change)**

Variables	30-Day return to ED	90-Day return to ED
Observation	0.508^a^ (0.0530)	1.04^a^ (0.0719)
Observation×black	0.454^a^ (0.156)	0.894^a^ (0.213)
Observation×other race	0.675^a^ (0.198)	0.949^a^ (0.270)
Observation×rural residence	−1.07^a^ (0.106)	−1.51^a^ (0.137)
Black	3.01^a^ (0.122)	6.06^a^ (0.174)
Other race	0.380^b^ (0.150)	0.749^a^ (0.208)
Rural residence	2.41^a^ (0.100)	3.67^a^ (0.134)
Age (in years)	0.0175^a^ (0.00306)	0.0975^a^ (0.00452)
Female	−0.0191 (0.0437)	0.406^a^ (0.0587)
Quan Index	1.13^a^ (0.0187)	2.68^a^ (0.0265)
Home w/services	5.49^a^ (0.0856)	10.7^a^ (0.115)
Skilled nursing facility	6.03^a^ (0.150)	12.0^a^ (0.198)
Other nursing home	1.64^a^ (0.128)	3.93^a^ (0.190)
Inpatient rehab facility	−1.98^a^ (0.244)	0.467 (0.357)
Hospice	−3.01^a^ (0.248)	−0.963^b^ (0.424)
Other	6.47^a^ (0.206)	8.86^a^ (0.254)
Weekend	0.186^a^ (0.0432)	0.189^a^ (0.0557)
Spring	−0.440^a^ (0.0509)	−0.200^a^ (0.0672)
Fall	−0.434^a^ (0.0545)	−0.335^a^ (0.0722)
Winter	−0.784^a^ (0.0542)	−0.656^a^ (0.0709)
2008	2.13^a^ (0.806)	1.22 (1.15)
2009	2.53^a^ (0.807)	1.86 (1.15)
2010	2.94^a^ (0.808)	2.54^b^ (1.16)
Constant	7.04^a^ (0.845)	10.6^a^ (1.21)
Observations	3,076,471	3,014,847
Hospital fixed effects	*n*=4725	*n*=4725

^a^ *p*<0.01.

^b^ *p*<0.05.

**Appendix Table A4.** **Linear Probability Model of Hospital Readmission (Percentage Point Change)**

Variables	30-Day readmissions	90-Day readmissions
Observation	−3.35^a^ (0.0571)	−4.27^a^ (0.0760)
Observation×black	−1.03^a^ (0.158)	−1.79^a^ (0.206)
Observation×other race	−1.33^a^ (0.194)	−2.33^a^ (0.273)
Observation×rural residence	−0.441^a^ (0.105)	−0.309^b^ (0.130)
Black	1.06^a^ (0.132)	2.68^a^ (0.163)
Other race	0.158 (0.157)	0.0447 (0.198)
Rural residence	0.709^a^ (0.0906)	0.932^a^ (0.111)
Age (in years)	0.0234^a^ (0.00278)	0.112^a^ (0.00396)
Female	−1.45^a^ (0.0420)	−2.17^a^ (0.0565)
Quan Index	2.55^a^ (0.0201)	4.81^a^ (0.0253)
Home w/services	8.45^a^ (0.101)	14.9^a^ (0.125)
Skilled nursing facility	13.0^a^ (0.170)	21.6^a^ (0.204)
Other nursing home	2.97^a^ (0.125)	6.37^a^ (0.172)
Inpatient rehab facility	3.84^a^ (0.298)	7.43^a^ (0.406)
Hospice	−3.64^a^ (0.264)	−2.38^a^ (0.431)
Other	9.02^a^ (0.246)	10.1^a^ (0.266)
Weekend	−0.115^a^ (0.0441)	−0.407^a^ (0.0564)
Spring	0.0411 (0.0500)	−0.175^a^ (0.0658)
Fall	−0.0792 (0.0564)	0.153^b^ (0.0730)
Winter	0.166^a^ (0.0542)	0.478^a^ (0.0691)
2008	0.466 (1.01)	0.503 (1.30)
2009	0.140 (1.00)	−0.0315 (1.30)
2010	−0.0668 (1.00)	−0.263 (1.30)
Constant	9.94^a^ (1.03)	12.4^a^ (1.33)
Observations	3,090,173	3,059,798
Hospital fixed effects	*n*=4725	*n*=4725

^a^ *p*<0.01.

^b^ *p*<0.05.

**Appendix Table A5.** **Linear Probability Model of Mortality (Percentage Point Change)**

Variables	30-Day mortality	90-Day mortality
Observation	−1.22^a^ (0.0265)	−1.50^a^ (0.0367)
Observation×black	0.427^a^ (0.0615)	0.396^a^ (0.0901)
Observation×other race	0.425^a^ (0.0817)	0.450^a^ (0.126)
Observation×rural residence	−0.00235 (0.0477)	−0.0824 (0.0658)
Black	−0.674^a^ (0.0509)	−0.945^a^ (0.0715)
Other race	−0.337^a^ (0.0660)	−0.570^a^ (0.101)
Rural residence	0.201^a^ (0.0407)	0.513^a^ (0.0538)
Age (in years)	0.0616^a^ (0.00132)	0.158^a^ (0.00203)
Female	−0.610^a^ (0.0170)	−1.50^a^ (0.0273)
Quan Index	1.19^a^ (0.0133)	2.86^a^ (0.0181)
Home w/services	1.34^a^ (0.0361)	4.05^a^ (0.0649)
Skilled nursing facility	7.98^a^ (0.114)	15.9^a^ (0.152)
Other nursing home	5.17^a^ (0.0855)	9.78^a^ (0.117)
Inpatient rehab facility	0.646^a^ (0.110)	2.31^a^ (0.202)
Hospice	64.0^a^ (0.411)	69.2^a^ (0.335)
Other	1.89^a^ (0.0931)	3.37^a^ (0.134)
Weekend	0.0105 (0.0184)	−0.0769^a^ (0.0274)
Spring	0.0230 (0.0212)	−0.116^a^ (0.0319)
Fall	0.124^a^ (0.0248)	0.261^a^ (0.0370)
Winter	0.242^a^ (0.0232)	0.309^a^ (0.0348)
2008	0.150 (0.425)	−0.0215 (0.658)
2009	0.100 (0.425)	−0.154 (0.658)
2010	0.106 (0.425)	−0.0917 (0.658)
Constant	−3.73^a^ (0.435)	−9.52^a^ (0.673)
Observations	3,155,958	3,155,958
Hospital fixed effects	*n*=4729	*n*=4729

^a^ *p*<0.01.

## References

[1] National Hospital Ambulatory Medical Care Survey: 2011 Emergency Department Summary Tables. 2014 Available at www.cdc.gov/nchs/data/ahcd/nhamcs_emergency/2011_ed_web_tables.pdf Accessed 1027, 2016

[2] Ross MA, Graff LG Principles of observation medicine. Emerg Med Clin North Am. 2001;19:1–17 11214392 10.1016/s0733-8627(05)70165-6

[3] Baugh CW, Venkatesh AK, Bohan JS Emergency department observation units: a clinical and financial benefit for hospitals. Health Care Manage Rev. 2011;36:28–37 21157228 10.1097/HMR.0b013e3181f3c035

[4] Ross MA, Aurora T, Graff L, et al. State of the art: emergency department observation units. Crit Pathw Cardiol. 2012;11:128–138 22825533 10.1097/HPC.0b013e31825def28

[5] Ross MA, Hockenberry JM, Mutter R, et al. Protocol-driven emergency department observation units offer savings, shorter stays, and reduced admissions. Health Aff (Millwood). 2013;32:2149–2156 24301399 10.1377/hlthaff.2013.0662

[6] Baugh CW, Venkatesh AK, Hilton JA, et al. Making greater use of dedicated hospital observation units for many short-stay patients could save $3.1 billion a year. Health Aff (Millwood). 2012;31:2314–2323 23019185 10.1377/hlthaff.2011.0926

[7] Kangovi S, Cafardi SG, Smith RA, et al. Patient financial responsibility for observation care. J Hosp Med. 2015;10:718–723 26292192 10.1002/jhm.2436

[8] Venkatesh AK, Geisler BP, Chambers JJG, et al. Use of observation care in US emergency departments, 2001 to 2008. PLoS One. 2011;6:e24326 21935398 10.1371/journal.pone.0024326 PMC3173457

[9] Feng Z, Wright B, Mor V Sharp rise in Medicare enrollees being held in hospitals for observation raises concerns about causes and consequences. Health Aff (Millwood). 2012;31:1251–1259 22665837 10.1377/hlthaff.2012.0129 PMC3773225

[10] Wright B, Jung HY, Feng Z, et al. Trends in observation care among Medicare fee‐for‐service beneficiaries at critical access hospitals, 2007–2009. J Rural Health. 2013;29 Suppl 1:s1–s6 23944275 10.1111/jrh.12007 PMC3752707

[11] Baicker K, Chandra A, Skinner JS, et al. Who you are and where you live: how race and geography affect the treatment of Medicare beneficiaries. Health Aff (Millwood). 2004;Suppl Variation:VAR33–VAR44 15471775 10.1377/hlthaff.var.33

[12] Jha AK, Orav EJ, Li Z, et al. Concentration and quality of hospitals that care for elderly black patients. Arch Intern Med. 2007;167:1177–1182 17563027 10.1001/archinte.167.11.1177

[13] Jha AK, Orav EJ, Epstein AM Low-quality, high-cost hospitals, mainly in South, care for sharply higher shares of elderly black, Hispanic, and Medicaid patients. Health Aff (Millwood). 2011;30:1904–1911 21976334 10.1377/hlthaff.2011.0027

[14] Smedley BD, Stith AY, Nelson AR Unequal Treatment: Confronting Racial and Ethnic Disparities in Health Care (with CD). Washington, DC: National Academies Press, 2009 25032386

[15] Charlson ME, Pompei P, Ales KL, et al. A new method of classifying prognostic comorbidity in longitudinal studies: development and validation. J Chronic Dis. 1987;40:373–383 3558716 10.1016/0021-9681(87)90171-8

[16] Quan H, Sundararajan V, Halfon P, et al. Coding algorithms for defining comorbidities in ICD-9-CM and ICD-10 administrative data. Med Care. 2005;43:1130–1139 16224307 10.1097/01.mlr.0000182534.19832.83

[17] Ashton CM, Del Junco DJ, Souchek J, et al. The association between the quality of inpatient care and early readmission: a meta-analysis of the evidence. Med Care. 1997;35:1044–1059 9338530 10.1097/00005650-199710000-00006

[18] Anderson GF, Steinberg EP Hospital readmissions in the Medicare population. N Engl J Med. 1984;311:1349–1353 6436703 10.1056/NEJM198411223112105

[19] Sheehy AM, Graf B, Gangireddy S, et al. Hospitalized but not admitted: characteristics of patients with “observation status” at an academic medical center. JAMA Intern Med. 2013;173:1991–1998 23835927 10.1001/jamainternmed.2013.8185 PMC3942156

[20] Roberts RR, Zalenski RJ, Mensah EK, et al. Costs of an emergency department—based accelerated diagnostic protocol vs hospitalization in patients with chest pain: a randomized controlled trial. JAMA. 1997;278:1670–1676 9388086

[21] Cafardi SG, Pines JM, Deb P, et al. Increased observation services in Medicare beneficiaries with chest pain. Am J Emerg Med. 2016;34:16–19 26490388 10.1016/j.ajem.2015.08.049

[22] Bellolio MF, Sangaralingham LR, Schilz SR, et al. Observation status or inpatient admission: impact of patient disposition on outcomes and utilization among emergency department patients with chest pain. Acad Emerg Med. 2017;24:152–160 27739128 10.1111/acem.13116

[23] Dharmarajan K, Qin L, Bierlein M, et al. Outcomes after observation stays among older adult Medicare beneficiaries in the USA: retrospective cohort study. BMJ. 2017;357:j2616 28634181 10.1136/bmj.j2616 PMC5476173

[24] Wright B, Jung HY, Feng Z, et al. Hospital, patient, and local health system characteristics associated with the prevalence and duration of observation care. Health Serv Res. 2014;49:1088–1107 24611617 10.1111/1475-6773.12166 PMC4111799

[25] Jha AK, Orav EJ, Zheng J, et al. The characteristics and performance of hospitals that care for elderly Hispanic Americans. Health Aff (Millwood). 2008;27:528–537 18332511 10.1377/hlthaff.27.2.528

[26] Napoli AM, Choo EK, Dai J, et al. Racial disparities in stress test utilization in an emergency department chest pain unit. Crit Pathw Cardiol. 2013;12:9–13 23411602 10.1097/HPC.0b013e31827c9a86

[27] Pines JM, Russell Localio A, Hollander JE Racial disparities in emergency department length of stay for admitted patients in the United States. Acad Emerg Med. 2009;16:403–410 19245372 10.1111/j.1553-2712.2009.00381.x

[28] Calder LA, Forster AJ, Stiell IG, et al. Mapping out the emergency department disposition decision for high-acuity patients. Ann Emerg Med. 2012;60:567–576.e4 22699018 10.1016/j.annemergmed.2012.04.013

[29] Rising KL, Padrez KA, O'Brien M, et al. Return visits to the emergency department: the patient perspective. Ann Emerg Med. 2015;65:377–386.e3 25193597 10.1016/j.annemergmed.2014.07.015

[30] Singhal A, Tien Y-Y, Hsia RY Racial-ethnic disparities in opioid prescriptions at emergency department visits for conditions commonly associated with prescription drug abuse. PLoS One. 2016;11:e0159224 27501459 10.1371/journal.pone.0159224 PMC4976905

[31] Tamayo-Sarver JH, Hinze SW, Cydulka RK, et al. Racial and ethnic disparities in emergency department analgesic prescription. Am J Public Health. 2003;93:2067–2073 14652336 10.2105/ajph.93.12.2067 PMC1448154

[32] Smulowitz PB, Barrett O, Hall MM, et al. Physician variability in management of emergency department patients with chest pain. West J Emerg Med. 2017;18:592–600 28611878 10.5811/westjem.2017.2.32747 PMC5468063

[33] Sleath B, Roter D, Chewning B, et al. Asking questions about medication: analysis of physician-patient interactions and physician perceptions. Med Care. 1999;37:1169–1173 10549619 10.1097/00005650-199911000-00009

[34] Green AR, Carney DR, Pallin DJ, et al. Implicit bias among physicians and its prediction of thrombolysis decisions for black and white patients. J Gen Intern Med. 2007;22:1231–1238 17594129 10.1007/s11606-007-0258-5 PMC2219763

[35] Richardson LD, Babcock Irvin C, Tamayo‐Sarver JH Racial and ethnic disparities in the clinical practice of emergency medicine. Acad Emerg Med. 2003;10:1184–1188 14597493 10.1111/j.1553-2712.2003.tb00601.x

[36] Wiler JL, Ross MA, Ginde AA National study of emergency department observation services. Acad Emerg Med. 2011;18:959–965 21883638 10.1111/j.1553-2712.2011.01151.x

